# Unraveling the Phase Stability and Physical Property of Modulated Martensite in Ni_2_Mn_1.5_In_0.5_ Alloys by First-Principles Calculations

**DOI:** 10.3390/ma15114032

**Published:** 2022-06-06

**Authors:** Xin-Zeng Liang, Jing Bai, Zi-Qi Guan, Yu Zhang, Jiang-Long Gu, Yu-Dong Zhang, Claude Esling, Xiang Zhao, Liang Zuo

**Affiliations:** 1 Key Laboratory for Anisotropy and Texture of Materials, Northeastern University, Shenyang 110819, China; 1810167@stu.neu.edu.cn (X.-Z.L.); 13383881381@163.com (Z.-Q.G.); 2110136@stu.neu.edu.cn (Y.Z.); lzuo@mail.neu.edu.cn (L.Z.); 2 School of Resources and Materials, Northeastern University at Qinhuangdao, Qinhuangdao 066004, China; 3 Hebei Provincial Laboratory for Dielectric and Electrolyte Functional Materials, Qinhuangdao 066004, China; 4 State Key Laboratory of Metastable Materials Science and Technology, Yanshan University, Qinhuangdao 066004, China; gujianglong@ysu.edu.cn; 5 Laboratoire d’Étude des Microstructures et de Mécanique des Matériaux, LEM3 CNRS, UMR 7239, University of Lorraine, 57045 Metz, France; yudong.zhang@univ-lorraine.fr (Y.-D.Z.); claude.esling@univ-lorraine.fr (C.E.)

**Keywords:** Ni–Mn–In, first-principles calculations, modulated martensite, Jahn–Teller effect

## Abstract

Large magnetic field-induced strains can be achieved in modulated martensite for Ni-Mn-In alloys; however, the metastability of the modulated martensite imposes serious constraints on the ability of these alloys to serve as promising sensor and actuator materials. The phase stability, magnetic properties, and electronic structure of the modulated martensite in the Ni_2_Mn_1.5_In_0.5_ alloy are systematically investigated. Results show that the 6M and 5M martensites are metastable and will eventually transform to the NM martensite with the lowest total energy in the Ni_2_Mn_1.5_In_0.5_ alloy. The physical properties of the incommensurate 7M modulated martensite (7M–IC) and nanotwinned 7M martensite ($7M-{\left(5\bar{2}\right)}_{2}$) are also calculated. The austenite (A) and $7M-{\left(5\bar{2}\right)}_{2}$ phases are ferromagnetic (FM), whereas the 5M, 6M, and NM martensites are ferrimagnetic (FIM), and the FM coexists with the FIM state in the 7M–IC martensite. The calculated electronic structure demonstrates that the splitting of Jahn–Teller effect and the strong Ni–Mn bonding interaction lead to the enhancement of structural stability.

## 1. Introduction

Ferromagnetic shape-memory alloys have attracted great interest due to their properties such as favorable magnetic field-induced strain (MFIS) and magnetocaloric effects (MCEs) [1,2,3,4,5]. Those properties are crucial to the utilization of Ni–Mn-based alloys in applications such as magnetic-driven actuators and solid-state energy-efficient refrigeration. The important factors for achieving large MFIS depend on the type of martensite structure with its c/a ratio around 1.00 [1,6,7,8,9]. For example, for modulated martensite with c/a < 1.00, 5.1% and 6% MFIS were obtained in the five layer modulated (5M) martensite [6,7] and 9.5% MFIS in the seven-layer modulated (7M) martensite [1] of the Ni–Mn–Ga alloys. Sozinov et al. [8] achieved a reduction in c/a value in the non-modulated (NM) martensite, from 1.25 [9] to 1.15, by co-doping Co and Cu in the Ni_2_MnGa alloy; thus, an MFIS as large as 12% could be obtained.

Austenite (A) can develop modulated (including 5M, six-layer modulated martensite (6M), and 7M) and non-modulated martensite (NM) structures after martensitic transformation in the Ni–Mn-based alloy [10,11,12,13]. The observed modulated martensite structures are mainly described by lattice modulation (including commensurate and incommensurate) and nanotwinning (long-period stacking order) [14,15]. The lattice modulation model gives the degree of deviation from equilibrium position for each atom in a periodically amplitude-modulated structure by a modulation equation, e.g., the monoclinic incommensurate model for 7M martensite (7M–IC) [14]; the long-range stacking order model assumes that the atoms in each plane are uniformly sheared, e.g., the

$${\left(5\bar{2}\right)}_{2}$$

stacking order for 7M martensite ($7M-{\left(5\bar{2}\right)}_{2}$) [15]. There has been a controversy over the two types of 7M–IC and $7M-{\left(5\bar{2}\right)}_{2}$ martensites due to the complexity of the long-period structure. A large number of experiments on these two types of 7M martensite have been performed [16,17,18,19,20,21,22,23,24,25].

The parent phase has an ordered L2_1_ structure in the Ni–Mn–In alloy and the martensitic transformation shows a non-diffusion type; the modulated martensitic structure in the Ni–Mn–Ga alloy is also extended to the Ni–Mn–In alloy. Righi et al. [23] and Kaufmann et al. [24] stated that the 7M martensite showed a monoclinic 7M–IC model and $7M-{\left(5\bar{2}\right)}_{2}$ nanotwin combination structure for the Ni–Mn–Ga alloy, respectively. Li et al. [25,26] confirmed the monoclinic commensurate structure of the 5M martensite and the monoclinic incommensurate structure of the 7M martensite from the EBSD Kikuchi diffraction patterns. The phase stability and magnetic properties of the commensurate 5M and 7M–IC were subsequently investigated by Xu et al. [27,28] using first-principles calculations based on the experimental results of Li et al.

Liang et al. [29,30] reported that the Ni_50_Mn_37.5_In_12.5_ alloy exhibited a 6M martensitic structure at room temperature (RT) by X-ray diffraction (XRD). Krenke et al. [31] determined the crystal structures of the Ni_0.5_Mn_0.5−x_In_x_ (0.05 ≤ x ≤ 0.25) alloys at RT by XRD. When x = 0.05, the alloy presented an NM martensite; for x = 0.10, the crystal structure of the alloy was a monoclinic 7M martensite; and the alloy possessed a monoclinic 5M structure for x = 0.15 and 0.155. Hernando et al. [32] indicated that the Ni_50_Mn_36_In_14_ alloy had a 5M martensite structure and the Mn_50_Ni_40_In_10_ alloy had a 7M martensitic structure by XRD at 150 K. Yan et al. [33] determined that the 6M martensite possessed a monoclinic incommensurate structure based on neutron diffraction and (3 + 1) D superspace theory in the Ni_2_Mn_1.44_In_0.56_ alloy.

Due to the complexity of the modulated structures, it is difficult to study the phase stability and magnetic properties of different modulated martensitic structures in experiments. Studying the physical properties of modulated martensites by first-principles calculations is a feasible approach. The main purpose of this work is to reveal the phase stability of the $7M-{\left(5\bar{2}\right)}_{2}$ and 7M–IC models existing in experiments by means of the first-principles calculations and to explain the physical nature of the phase stability from the electronic structure. Meanwhile, the austenite (A), 5M, 6M, and NM structures are also taken into account in order to systematically investigate the possible phases experimentally observed in the Ni–Mn–In alloy. This study attempts to comprehend the two experimentally disputed modulation models from a thermodynamic standpoint and provides theoretical support for further research.

## 2. Computational Methods

**Figure 1:**
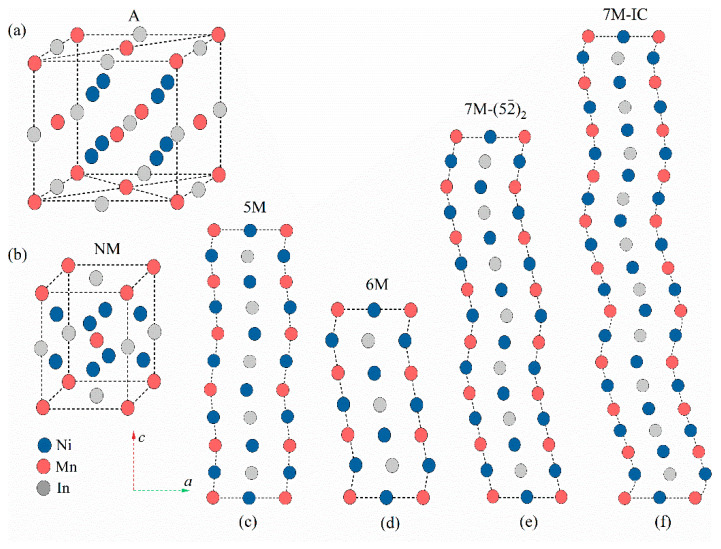
Crystal structures of (**a**) cubic austenite, (**b**) NM martensite, (**c**) 5M martensite, (**d**) 6M martensite, (**e**) $7M-{\left(5\bar{2}\right)}_{2}$ martensite, and (**f**) 7M–IC martensite for Ni_2_MnIn alloy.

The presented calculations were performed with the spin-polarized density-functional theory (DFT) as implemented by the Vienna ab initio Simulation Package (VASP) [34]. The interaction between ions and electrons was described by the projector augmented wave (PAW) method [35], and the exchange–correlation potential was described using the Perdew–Burke–Ernzerhof implementation of a generalized gradient approximation (GGA) [36]. Ni-3*d*^8^4*s*^2^, Mn-3*d*^5^4*s^2^*, and In-4*d*^10^5*s*^2^5*p* were treated as valence states. The cutoff energy of the plane waves was set to 351 eV. The Brillouin zone was sampled by the Monkhorst–Pack grid [37] with a 10 × 10 × 10 *k*-point mesh for the A structure, a 7 × 11 × 5 mesh for the 6M structure, an 8 × 6 × 4 mesh for the 5M and 7M structures, and a 7 × 14 × 10 mesh for the NM structure. Due to the difference in the initial lattice constants of the different martensitic structures, the *k*-point mesh was different based on the Brillouin zone and lattice constants of the austenitic phase. The total energy convergence criterion was set to 10^−3^ eV and the total and atomic forces were set to 0.02 eV/Å for all calculations. For the A and NM structures, 16-atom cells were created, and 40-atom, 24-atom, 56-atom, and 80-atom unit cells were established for the 5M, 6M, $7M-{\left(5\bar{2}\right)}_{2}$, and 7M–IC structures, respectively. The crystal structure model is shown in Figure 1. It should be noted that the modulated martensite models were based on the experimentally resolved structures. Schematic diagrams and detailed atomic Wyckoff positions of the modulated structures involved here are given in Figure S1 and Tables S1–S4 of the supplementary material. The ferromagnetic (FM) and ferrimagnetic (FIM) states were considered for all possible phases; details can be found in Figure S2 of the Supplementary Materials.

## 3. Results and Discussion

### 3.1. Structural Parameters of Possible Phases

Table 1 shows the equilibrium lattice constants in the FM and FIM states for the possible phases of the Ni_2_Mn_1.5_In_0.5_ alloy. Our calculated result for the A phase in the FM state is 5.95 Å, which is in excellent accordance with the previous theoretical values (5.962 Å [38] and 5.95 Å [39]). Because there is no available experimental evidence for the alloy with the same composition, the XRD results at RT for the Ni_2_Mn_1.52_In_0.48_ and Ni_2_Mn_1.48_In_0.52_ alloys were chosen for comparison with the calculated results for the 7M martensite. The experimental values are relatively close to those obtained from the $7M-{\left(5\bar{2}\right)}_{2}$ structure. However, because the lattice constants are affected by the alloy composition, temperature, and the macroscopic strain field present in the martensite, it remains uncertain which modulated martensite will ultimately be realized in the alloy. Another noteworthy point is that for each structure, the crystal volume (*V*) of the FM state is larger than that of the FIM state. This is due to the magnetic factor as was noted earlier: the lattice constant of the FM state is greater than that of the non-ferromagnetic state [40].

The optimized lattice constants for the 6M martensite in the FIM state agree with the experimental value measured at T = 300 K using the conventional least-squares approach [29,41]. In particular, the relative error between the theoretically calculated lattice constants for the 6M martensite in the FIM state and those experimentally measured by Wang et al. [41] is only 0.13~0.77%.

**Table 1:** Theoretical lattice parameters of possible phases of Ni_2_Mn_1.5_In_0.5_ alloy in FM and FIM states in comparison with experimental or other theoretical data.

Structure	Lattice Parameter					
		*a* (Å)	*b* (Å)	*c* (Å)	*β* (Å)	*V*
A	FM	5.95, 5.962 ^a^, 5.95 ^b^			90	52.57
	FIM	5.93, 5.94 ^a^			90	52.15
5M	FM	4.21	5.91	21.05	90.17	52.42
	FIM	4.41	5.49	21.30	89.03	51.64
6M	FM	4.26	5.82	12.71	91.40	52.51
	FIM	4.41	5.47	12.89	94.07	51.70
	Exp. ^c^	4.66	5.40	12.80	95.24	53.49
	Exp. ^d^	4.42	5.48	12.99	94.19	
$7M-(5\bar{2}$)_2_	FM	4.23	5.88	29.55	92.01	52.44
	FIM	4.37	5.55	30.05	95.45	51.78
7M–IC	FM	4.24	5.87	42.28	91.14	52.63
	FIM	4.39	5.52	43.12	94.89	52.02
	Exp. ^e^	4.37	5.69	30.21	93.67	
	Exp. ^e^	4.35	5.73	30.38	93.24	
NM	FM	4.21		5.95	90	52.64
	FIM	3.87		6.89	90	51.49

^a^ Ref. [38], EMTO-CPA. ^b^ Ref. [39], GGA-PBE ^c^ Ref. [29], XRD. ^d^ Ref. [41], XRD. ^e^ Ref. [42], XRD.

### 3.2. Phase Stability of Possible Phases

**Figure 2:**
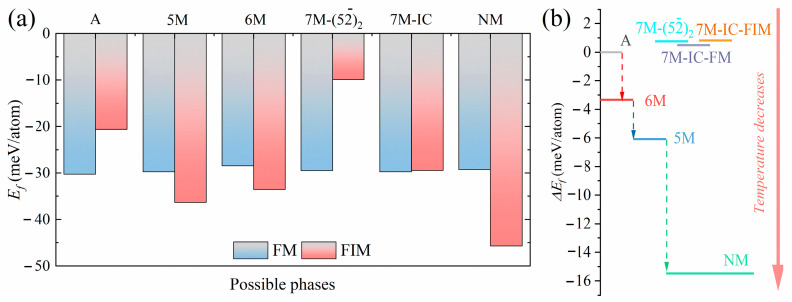
(**a**) Formation energy of each of the possible phases in FM and FIM states, (**b**) formation energy difference between each possible martensitic phase and austenite of Ni_2_Mn_1.5_In_0.5_ alloy.

To determine the phase stability of each possible phase in the Ni_2_Mn_1.5_In_0.5_ alloy, the formation energies in the FM and FIM states were calculated and the results are shown in Figure 2a. The formation energy can be calculated as previously reported [43].

As can be seen from Figure 2a, for both the A and $7M-{\left(5\bar{2}\right)}_{2}$ phases, the formation energy in the FM state is lower than that in the FIM state, indicating that the A and $7M-{\left(5\bar{2}\right)}_{2}$ phases are more likely to possess the FM state; whereas for the 7M–IC martensite, the difference in formation energy between the FM and FIM states is small, only about 0.32 meV/atom, implying that the 7M–IC martensite is strongly susceptible to the co-existence of the FM and FIM states due to incomplete Curie transformation of the martensite. The magnetic ground state of the 7M–IC martensite below is considered to be the FM state for convenience. The formation energy of the FIM state is lower than that of the FM state for the 5M, 6M, and NM martensites, implying that these martensites display the FIM state.

The formation energy difference between austenite and different martensites is also calculated based on the determination of each phase’s magnetic ground state; the results are shown in Figure 2b. The formation energies of the two models of the 7M martensite are almost equal, with a difference of only 0.06 meV/atom between the $7M-{\left(5\bar{2}\right)}_{2}$ and 7M–IC in the FM state. This means that the difference in phase stability between these two phases is not significant. It is probable that the macroscopic stress field during the martensitic transformation determines which model of the 7M modulated structure is presented in the experiments. This may be one of the reasons for the controversy between the two models in the experiments. Notice that the reasons for contradictory experimental observations could be also different. For example, it was shown for the 5M martensite in the Ni–Mn–Ga alloys that modulation periodicity changes from commensurate to incommensurate with the decrease in temperature and is accompanied by the refinement of the *a/b* laminate [44,45,46]. We also found that the formation energy of the 7M martensite is 0.5 meV/atom higher than that of the A phase. This indicates that the 7M martensite is not transformed from the A phase by a thermodynamic driving force. However, the 7M martensite observed in the experiments is likely to be induced by the local stress concentration. For the other martensites, the difference in formation energy is more pronounced. In previous experiments, it was observed that the Ni_2_Mn_1.5_In_0.5_ alloy exhibited 6M martensite at RT [30].

**Figure 3:**
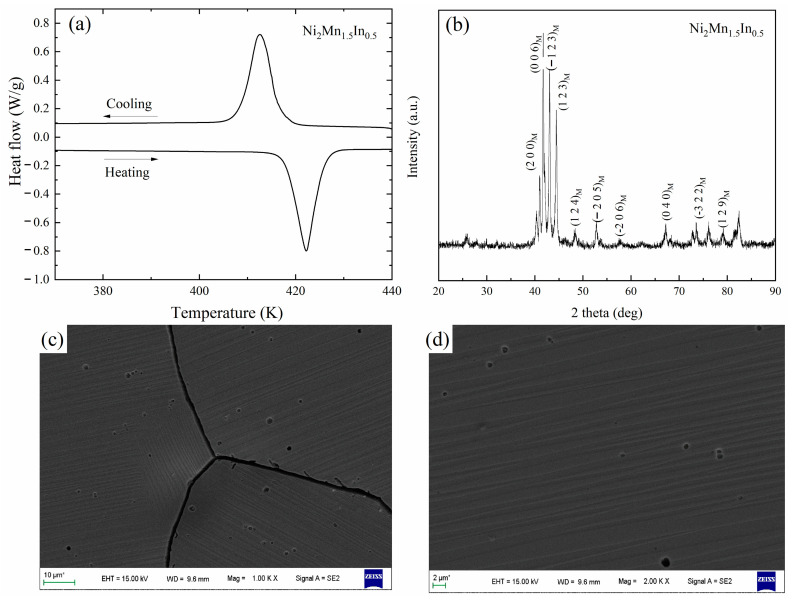
(**a**) DSC curve [30], (**b**) XRD pattern at room temperature [30], (**c**,**d**) microstructures of Ni_2_Mn_1.5_In_0.5_ alloy.

The Ni_2_Mn_1.5_In_0.5_ alloy sample has been previously melted and experimentally characterized by DSC [30], XRD [30], and SEM. The results are shown in Figure 3. As can be seen from Figure 3, the martensitic transformation is observed, and the martensitic transformation temperatures are M_s_ = 408 K, M_f_ = 417 K, A_s_ = 416 K, and A_f_ = 426 K, respectively. The SEM results show that the alloy presents slatted modulated martensite at room temperature. Furthermore, the martensitic laths have different orientations and different thicknesses in different grains, indicating that different types of martensite may coexist in the Ni_2_Mn_1.5_In_0.5_ alloy at room temperature. The XRD curve of the Ni_2_Mn_1.5_In_0.5_ alloy shows the 6M modulated martensitic structure at room temperature, which is consistent with the calculated results. Combining the results of the first-principles calculations and experiments, it can be seen that the 6M and 5M modulated martensitic structures are metastable; the NM martensite is the most stable structure of the Ni_2_Mn_1.5_In_0.5_ alloy. As confirmed by Dutta et al., the lowest energy structure of martensite is the NM martensite [47].

### 3.3. Total/Atomic Magnetic Moment

**Figure 4:**
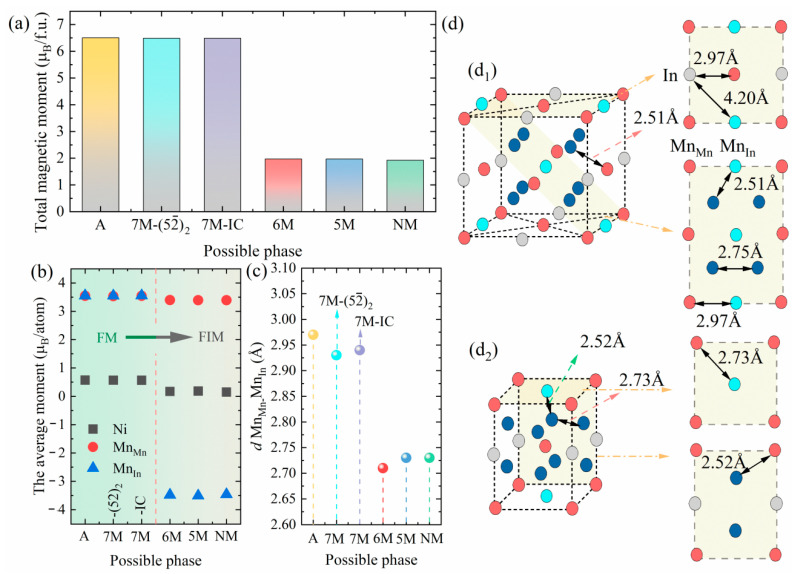
(**a**) Total magnetic moment, (**b**) average of Ni, Mn_Mn_, and Mn_In_ moments, (**c**) nearest-neighbor atomic distance between Mn_Mn_ and Mn_In_ atoms of Ni_2_Mn_1.5_In_0.5_ alloy, and (**d**) schematic diagram of the nearest atomic distance for (**d_1_**) A phase and (**d_2_**) NM phase.

The total and atomic magnetic moments of the possible phases in the Ni_2_Mn_1.5_In_0.5_ alloy are shown in Figure 4a,b, respectively. The total magnetic moments of the A, $7M-{\left(5\bar{2}\right)}_{2}$, and 7M–IC phases in the FM state have little difference. The total magnetic moment of the A phase is about 6.51 *μ*_B_/f.u., which agrees well with the literature values (6.4 *μ*_B_/f.u. [38] and 6.5 *μ*_B_/f.u. [39]). The total magnetic moment decreases abruptly as A transforms to the 6M martensite, indicating that a magnetostructural coupling transformation occurs. The magnetostructural coupling can increase not only the MFIS [48], but also the magnetization difference ∆*M* [49], thus making such material appealing as a magnetomechanical actuator. The total magnetic moments of the 6M, 5M, and NM are almost the same. The trend in the Ni atomic moment is consistent with the trend in the total magnetic moment, and the magnetic moments of the excess Mn_In_ atoms in the 6M, 5M, and NM phases are all negative, indicating the spin direction of Mn_Mn_ and Mn_In_ present an antiparallel alignment.

To investigate the underlying reason for the change in the magnetic ground state of each phase, we calculated the nearest-neighbor atomic distances in the A and NM phases, as shown in Figure 4d. It can be seen that the atomic distances of Ni-Mn_Mn_, Ni-Mn_In_, and Ni-Ni remain almost constant during the A→NM transformation (2.51, 2.51, 2.75 Å for the A phase and 2.52, 2.52, 2.73 Å for the NM phase, respectively); whereas the Mn_Mn_-Mn_In_ atomic distance (*d* (Mn_Mn_-Mn_In_)) decreases from 2.97 Å to 2.73 Å. The *d* (Mn_Mn_-Mn_In_) for the possible phases are summarized in Figure 4c. This indicates that the shortening of *d* (Mn_Mn_-Mn_In_) leads to enhanced interaction between the Mn_Mn_ and Mn_In_ atoms, resulting in a magnetostructural coupling transformation.

### 3.4. Electronic Structure

**Figure 5:**
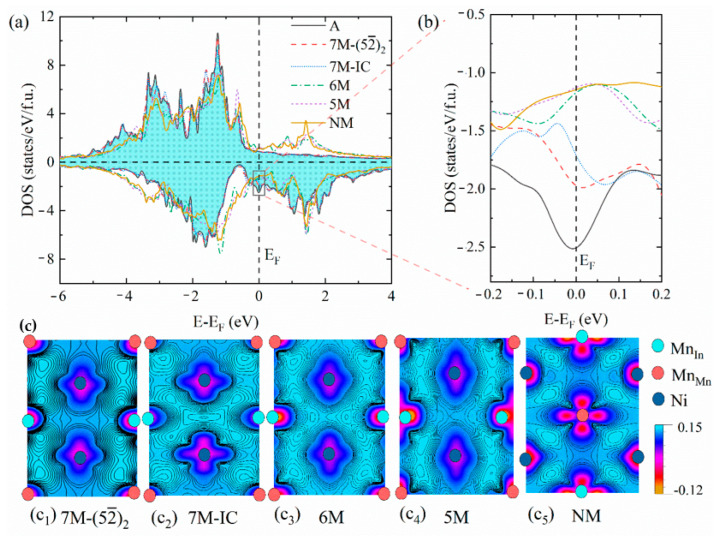
(**a**) Total density of states of A, 5M, 6M, $7M-{\left(5\bar{2}\right)}_{2}$, 7M–IC, and NM phases of Ni_2_Mn_1.5_In_0.5_ alloy, (**b**) enlarged spin-down density of states near the E_F_, and (**c**) differential charge densities of different martensitic structures in plane with excess Mn_In_ atoms.

To understand the physical nature of the relative stability of each martensite, the total densities of states (DOS) and the differential charge densities of the possible phases are shown in Figure 5. The relative stability of the different structures can be understood not only by the features near the Fermi level (E_F_) [50,51,52], but can also be influenced by the bonding ability between Ni and Mn [27,53].

It can be seen from Figure 5a that the main change in the density of states is concentrated near the E_F_. The total DOS near the E_F_ is enlarged, as shown in Figure 5b. The peak of the A phase is located exactly at the E_F_, and the spin-down density of states at the E_F_ is the largest. This indicates that the A phase is extremely unstable. As the symmetry decreases, the states at the E_F_ are redistributed due to martensitic transformation. The $7M-{\left(5\bar{2}\right)}_{2}$ and 7M–IC martensites also have peaks near the E_F_, but their numbers of states are lower than that of the A phase. However, for the 6M, 5M, and NM martensites, the pseudopotential valleys appear at the E_F_. This suggests that a Jahn–Teller effect [54,55,56] occurs in the alloy as the martensitic transformation takes place, which stabilizes the 6M, 5M, and NM martensites.

As can be seen in Figure 5c, the bonding behavior not only exists between Ni-Mn_Mn_, but also for Ni-Mn_In_ for all the martensitic structures. The bonding ability of Ni-Mn_Mn_(Mn_In_) in the modulated martensite is not significantly different. However, the bonding ability between Ni-Mn_Mn_(Mn_In_) in the NM martensite is stronger than that in the modulated martensite. Therefore, the bonding ability between Ni and Mn also plays an important role in phase stability.

## 4. Conclusions

Based on first-principles calculations, a comprehensive study of the structural and electronic properties of the Ni_2_Mn_1.5_In_0.5_ alloy was carried out. The phase stability and magnetic properties of the experimentally observed 5M, 6M, 7M–IC, $7M-{\left(5\bar{2}\right)}_{2}$, and NM martensitic structures were investigated. The calculated equilibrium lattice constants are in good agreement with those determined by experiments and theoretical calculations. For the 7M martensite, the formation energies of the two models are very close. The A and $7M-{\left(5\bar{2}\right)}_{2}$ phases possess FM states; the FM and FIM states co-exist in the 7M–IC martensite; and the 5M, 6M, and NM martensites prefer to exhibit the FIM states. The alloy undergoes a magnetostructural coupling transformation, which is attributed to the shortening of the Mn_Mn_-Mn_In_ atomic distance. The phase stability is dependent on the Jahn–Teller effect and the bonding behavior between Ni and Mn.

## Data Availability

The data that support the findings of this study are available from the corresponding author upon reasonable request.

## Supplementary Materials

The following supporting information can be downloaded at: https://www.mdpi.com/article/10.3390/ma15114032/s1, see Supplementary Materials for the schematic diagrams and detailed atomic Wyckoff positions of the modulated structures and the setting of magnetic configurations. References [20,23,33,57] is cited in the supplementary materials.

## References

[1] Sozinov A., Likhachev A., Lanska N., Ullakko K. (2002). Giant magnetic-field-induced strain in NiMnGa seven-layered martensitic phase. Appl. Phys. Lett..

[2] Chmielus M., Zhang X., Witherspoon C., Dunand D., Müllner P. (2009). Giant magnetic-field-induced strains in polycrystalline Ni-Mn-Ga foams. Nat. Mater..

[3] Ullakko K., Huang J., Kantner C., Ohandley R., Kokorin V. (1996). Large magnetic-field-induced strains in Ni_2_MnGa single crystals. Appl. Phys. Lett..

[4] Yang J., Li Z., Yang B., Yan H., Cong D., Zhao X., Zuo L. (2022). Effects of Co and Si co-doping on magnetostructural transformation and magnetocaloric effect in Ni-Mn-Sn based alloys. J. Alloys Comp..

[5] Pfeuffer L., Lemke J., Shayanfar N., Riegg S., Koch D., Taubel A., Scheibel F., Kani N.A., Adabifiroozjaei E., Molina-Luna L. (2021). Microstructure engineering of metamagnetic Ni-Mn-based Heusler compounds by Fe-doping: A roadmap towards excellent cyclic stability combined with large elastocaloric and magnetocaloric effects. Acta Mater..

[6] Heczko O., Sozinov A., Ullakko K. (2000). Giant field-induced reversible strain in magnetic shape memory NiMnGa alloy. IEEE Trans. Magn..

[7] Murray S.J., Marioni M., Allen S.M., O’Handley R.C., Lograsso T.A. (2000). 6% magnetic-field-induced strain by twin-boundary motion in ferromagnetic Ni-Mn-Ga. Appl. Phys. Lett..

[8] Sozinov A., Lanska N., Soroka A., Zou W. (2013). 12% magnetic field-induced strain in Ni-Mn-Ga-based non-modulated martensite. Appl. Phys. Lett..

[9] Yan H.L., Liu H.X., Zhao Y., Jia N., Bai J., Yang B., Li Z.B., Zhang Y.D., Esling C., Zhao X. (2021). Impact of B alloying on ductility and phase transition in the Ni–Mn-based magnetic shape memory alloys: Insights from first-principles calculation. J. Mater. Sci. Technol..

[10] Yan H.L., Huang X.M., Esling C. (2022). Recent Progress in Crystallographic Characterization, Magnetoresponsive and Elastocaloric Effects of Ni-Mn-In-Based Heusler Alloys—A Review. Front. Mater..

[11] Akır A.C., Righi L., Albertini F., Acet M., Farle M., Akturk S. (2013). Extended investigation of intermartensitic transitions in Ni-Mn-Ga magnetic shape memory alloys: A detailed phase diagram determination. J. Appl. Phys..

[12] Singh S., Petricek V., Rajput P., Hill A.H., Suard E., Barman S.R., Pandey D. (2014). High-resolution synchrotron x-ray powder diffraction study of the incommensurate modulation in the martensite phase of Ni_2_MnGa: Evidence for nearly 7M modulation and phason broadening. Phys. Rev. B.

[13] Wang H., Li D., Zhang G., Li Z., Yang B., Yan H., Cong D., Esling C., Zhao X., Zuo L. (2022). Highly sensitive elastocaloric response in a directionally solidified Ni_50_Mn_33_In_15. 5_Cu_1. 5_ alloy with strong <001> A preferred orientation. Intermetallics.

[14] Martynov V.V., Kokorin V.V. (1992). The crystal structure of thermally-and stress-induced martensites in Ni_2_MnGa single crystals. J. Phys. III.

[15] Otsuka K., Ohba T., Tokonami M., Wayman C.M. (1993). New description of long period stacking order structures of martensites in β phase alloys. Scr. Metall. Mater..

[16] Brown P.J., Crangle J., Kanomata T., Matsmuoto M., Neumann K.U., Ouladdiaf B., Ziebeck K.R.A. (2002). The crystal structure and phase transitions of the magnetic shape memory compound Ni_2_MnGa. J. Phys. Condens. Matter.

[17] Mariager S., Huber T., Ingold G. (2014). The incommensurate modulations of stoichiometric Ni_2_MnGa. Acta Mater..

[18] Righi L., Albertini F., Calestani G., Pareti L., Paoluzi A., Ritter C., Algarabel P.A., Morellon L., Ricardo Ibarra M. (2006). Incommensurate modulated structure of the ferromagnetic shape-memory Ni_2_MnGa martensite. J. Solid State Chem..

[19] Righi L., Albertini F., Pareti L., Paoluzi A., Calestani G. (2007). Commensurate and incommensurate “5M” modulated crystal structures in Ni-Mn-Ga martensitic phases. Acta Mater..

[20] Glavatskyy I., Glavatska N., Urubkov I., Hoffman J.U., Bourdarot F. (2008). Crystal and magnetic structure temperature evolution in Ni-Mn-Ga magnetic shape memory martensite. Mater. Sci. Eng. A.

[21] Gruner M.E., Fahler S., Entel P. (2014). Magnetoelastic coupling and the formation of adaptive martensite in magnetic shape memory alloys. Phys. Status Solodi B.

[22] Zayak A.T., Entel P., Enkovaara J., Ayuela A., Nieminen R.M. (2003). First-principles investigations of homogeneous lattice-distortive strain and shuffles in Ni_2_MnGa. J. Phys. Condens. Matter.

[23] Righi L., Albertini F., Villa E., Paoluzi A., Calestani G., Chernenko V., Besseghini S., Ritter C., Passaretti F. (2008). Crystal structure of 7M modulated Ni-Mn-Ga martensitic phase. Acta Mater..

[24] Kaufmann S., Rößler U.K., Heczko O., Wuttig M., Buschbeck J., Schultz L., Fähler S. (2010). Adaptive Modulations of Martensites. Phys. Rev. Lett..

[25] Li Z.B., Zhang Y.D., Esling C., Zhao X., Zuo L. (2011). Twin relationships of 5M modulated martensite in Ni-Mn-Ga alloy. Acta Mater..

[26] Li Z.B., Zhang Y.D., Esling C., Zhao X., Zuo L. (2011). Determination of the orientation relationship between austenite and incommensurate 7M modulated martensite in Ni-Mn-Ga alloys. Acta Mater..

[27] Xu N., Raulot J.M., Li Z.B., Zhang Y.D., Bai J., Peng W., Meng X.Y., Zhao X., Zuo L., Esling C. (2014). Composition dependent phase stability of Ni-Mn-Ga alloys studied by ab initio calculations. J. Alloys Comp..

[28] Bai J., Wang J.L., Shi S.F., Raulot J.M., Zhang Y.D., Esling C., Zhao X., Zuo L. (2019). Complete martensitic transformation sequence and magnetic properties of non-stoichiometric Ni_2_Mn_1.2_Ga_0.8_ alloy by first-principles calculations. J. Magn. Magn. Mater..

[29] Liang X.Z., Bai J., Gu J.L., Wang J.L., Yan H.L., Zhang Y.D., Esling C., Zhao X., Zuo L. (2020). Ab initio-based investigation of phase transition path and magnetism of Ni-Mn-In alloys with excess Ni or Mn. Acta Mater..

[30] Liang X.Z., Bai J., Gu J.L., Yan H.L., Zhang Y.D., Esling C., Zhao X., Zuo L. (2020). Probing martensitic transformation, kinetics, elastic and magnetic properties of Ni_2-x_Mn_1.5_In_0.5_Co_x_ alloys. J. Mater. Sci. Technol..

[31] Krenke T., Acet M., Wassermann E.F., Moya X., Manosa L., Planes A. (2006). Ferromagnetism in the austenitic and martensitic states of Ni-Mn-In alloys. Phys. Rev. B.

[32] Hernando B., Llamazares J.L.S., Santos J.D., Sanchez M.L., Escoda L., Sunol J.J., Varga R., Garcia C., Gonzalez J. (2009). Grain oriented NiMnSn and NiMnIn Heusler alloys ribbons produced by melt spinning: Martensitic transformation and magnetic properties. J. Magn. Magn. Mater..

[33] Yan H.L., Zhang Y.D., Xu N., Senyshyn A., Brokmeier H.G., Esling C., Zhao X., Zuo L. (2015). Crystal structure determination of incommensurate modulated martensite in Ni-Mn-In Heusler alloys. Acta Mater..

[34] Kresse G., Joubert D. (1999). From ultrasoft pseudopotentials to the projector augmented-wave method. Phys. Rev. B.

[35] Blochl P.E. (1994). Projector augmented-wave method. Phys. Rev. B Condens. Matter.

[36] Perdew J.P., Burke K., Ernzerhof M. (1996). Generalized gradient approximation made simple. Phys. Rev. Lett..

[37] Monkhorst H.J., Pack J.D. (1976). Special points for Brillouin-zone integrations. Phys. Rev. B.

[38] Li C.M., Luo H.B., Hu Q.M., Yang R., Johansson B., Vitos L. (2012). Role of magnetic and atomic ordering in the martensitic transformation of Ni-Mn-In from a first-principles study. Phys. Rev. B.

[39] He W.Q., Huang H.B., Liu Z.H., Ma X.Q. (2017). First-principles investigation of magnetic properties and metamagnetic transition of NiCoMnZ (Z = In, Sn, Sb) Heusler alloys. Intermetallics.

[40] Li C.M., Luo H.B., Hu Q.M., Yang R., Johansson B., Vitos L. (2010). First-principles investigation of the composition dependent properties of Ni_2+x_Mn_1-x_Ga shape-memory alloys. Phys. Rev. B.

[41] Wang C.H. (2015). Crystal Structure, Martensitic Transformation and Microstructure in Ni-Mn-In Meta-Magnetic Shape Memory Alloys. Master’s Thesis.

[42] Feng Y. (2009). Martensitic Transformation Behavior and Structure and Properties of Ni-Mn-In Based Alloys. Ph.D. Thesis.

[43] Xu N., Raulot J.M., Li Z.B., Bai J., Zhang Y.D., Zhao X., Zuo L., Esling C. (2012). Oscillation of the magnetic moment in modulated martensites in Ni_2_MnGa studied by ab initio calculations. Appl. Phys. Lett..

[44] Straka L., Drahokoupil J., Vertat P., Kopecek J., Zeleny M., Seiner H., Heczko O. (2017). Orthorhombic intermediate phase originating from {110} nanotwinning in Ni_50.0_Mn_28.7_Ga_21.3_ modulated martensite. Acta. Mater..

[45] Straka L., Drahokoupil J., Vertat P., Zeleny M., Kopecek J., Sozinov A., Heczko O. (2018). Low temperature a/b nanotwins in Ni_50_Mn_25+x_Ga_25-x_ Heusler alloys. Sci. Rep..

[46] Vertat P., Seiner H., Straka L., Klicpera M., Sozinov A., Fabelo O., Heczko O. (2021). Hysteretic structural changes within five-layered modulated 10M martensite o fNi–Mn–Ga (–Fe). J. Phys. Condens. Matter.

[47] Dutta B., Cakir A., Giacobbe C., Al-Zubi A., Hickel T., Acet M., Neugebauer J. (2016). Ab initio prediction of martensitic and intermartensitic phase boundaries in Ni-Mn-Ga. Phys. Rev. Lett..

[48] Marcos J., Planes A., Mañosa L., Casanova F., Batlle X., Labarta A., Martínez B. (2002). Magnetic field induced entropy change and magnetoelasticity in Ni-Mn-Ga alloys. Phys. Rev. B.

[49] Wu Z., Liu Z., Yang H., Liu Y., Wu G. (2011). Effect of Co addition on martensitic phase transformation and magnetic properties of Mn_50_Ni_40-x_In_10_Co_x_ polycrystalline alloys. Intermetallics.

[50] Kundu A., Gruner M.E., Siewert M., Hucht A., Entel P., Ghosh S. (2017). Interplay of phase sequence and electronic structure in the modulated martensites of Mn_2_NiGa from first-principles calculations. Phys. Rev. B.

[51] Ghosh S., Ghosh S. (2019). Role of composition, site ordering, and magnetic structure for the structural stability of off-stoichiometric Ni_2_MnSb alloys with excess Ni and Mn. Phys. Rev. B.

[52] Kundu A., Ghosh S. (2018). Site occupancy, composition and magnetic structure dependencies of martensitic transformation in Mn_2_Ni_1+x_Sn_1-x_. J. Phys. Condens. Matter.

[53] Liang X.Z., Jiang X.J., Gu J.L., Bai J., Guan Z.Z., Li Z.Z., Yan H.L., Zhang Y.D., Esling C., Zhao X. (2021). 5M and 7M martensitic stability and associated physical properties in Ni_50_Mn_35_In_15_ alloy: First-principles calculations and experimental verification. Scr. Mater..

[54] Barman S.R., Banik S., Chakrabarti A. (2005). Structural and electronic properties of Ni_2_MnGa. Phys. Rev. B.

[55] Liang Z., Li Q., Sun K., Luo H.J. (2019). Martensitic transition and magnetic structure in Zn-doped Heusler alloy Mn_2_NiGa: A theoretical approach. Phys. Chem. Solids.

[56] Zelený M., Sozinov A., Straka L., Björkman T., Nieminen R.M. (2014). First-principles study of Co- and Cu-doped along the tetragonal deformation path. Phys. Rev. B.

[57] Li Z.B. (2011). Study on Crystallographic Features of Ni-Mn-Ga Ferromagnetic Shape Memory Alloys. Ph.D. Thesis.

