# Supplementary material for: Unraveling the Phase Stability and Physical Property of Modulated Martensite in Ni2Mn1.5In0.5 Alloys by First-Principles Calculations
Source: Materials (Basel). 2022 Jun 6;15(11):4032. doi: 10.3390/ma15114032 (PMC9182368; doi:10.3390/ma15114032)
Supplement: Supplementary file 1 [file materials-15-04032-s001.zip › materials-1717993-supplementary.pdf]

Unraveling the phase stability and physical property of modulated martensite in $Ni_2Mn_{1.5}In_{0.5}$ alloys by first-principles calculations

Xinzeng Liang[1], Jing Bai[1,2,4,*], Ziqi Guan[1], Yu Zhang[1], Jianglong Gu[3], Yudong Zhang[5], Claude Esling[5], Xiang Zhao[1,*], Liang Zuo[1]

[1] Key Laboratory for Anisotropy and Texture of Materials, Northeastern University, Shenyang 110819, China

[2] School of Resources and Materials, Northeastern University at Qinhuangdao, Qinhuangdao 066004, China

[3] State Key Laboratory of Metastable Materials Science and Technology, Yanshan University, Qinhuangdao 066004, P.R. China

[4] Hebei Provincial Laboratory for Dielectric and Electrolyte Functional Materials, Qinhuangdao 066004, China

[5] Laboratoire d'Étude des Microstructures et de Mécanique des Matériaux, LEM3 CNRS, UMR 7239, University of Lorraine, 57045 Metz, France

*Corresponding author: baijing@neuq.edu.cn (Jing Bai), zhaox@mail.neu.edu.cn (Xiang Zhao)

1. Computational details

Fig. S1 shows the crystal structure of the 5M, 6M, 7M-$(5\bar{2})_2$, and 7M-IC modulated martensites in the $Ni_2Mn_{1.5}In_{0.5}$ alloy. The crystal structures of the $Ni_2Mn_{1.5}In_{0.5}$ alloy were built on the basis of the crystal structure of the Ni_2MnIn alloy by directly replacing In atoms with excess Mn atoms. Notice that we use the long-range chemical disorder of excess Mn atoms here.

The magnetic state of ferromagnetic state (FM) and ferrimagnetic state (FIM) can be described by the following Fig. S2. The user manual of the VASP software states that antiferromagnetic calculations can be performed by specifying anti-parallel magnetic moments for the atoms in the cell. In the calculation process, we set the magnetic moment direction of each atom to the same direction to calculate the FM state. To calculate the FIM state, the magnetic moment of the excess Mn atoms or Mn atoms that are not at their original positions are setting to the opposite direction to the normal Mn moments. Therefore, we do spin polarized calculations by setting the ISPIN = 2 in the INCAR file. Meanwhile, the initial magnetic moment including direction of each atom is specified in the MAGMOM item in the INCAR file. For example, MAGMOM = 8*2 4*5 2*5 2*1 and MAGMOM = 8*2 4*5 2*-5 2*1 for the FM and FIM states of the austenite in the $Ni_8Mn_6In_2$ alloy, respectively.

Tables S1-S4 show the fractional coordinates and the corresponding initial lattice parameters for

the 5M, 6M, 7M-$(5\bar{2})_2$, and 7M-IC modulated martensites, respectively.

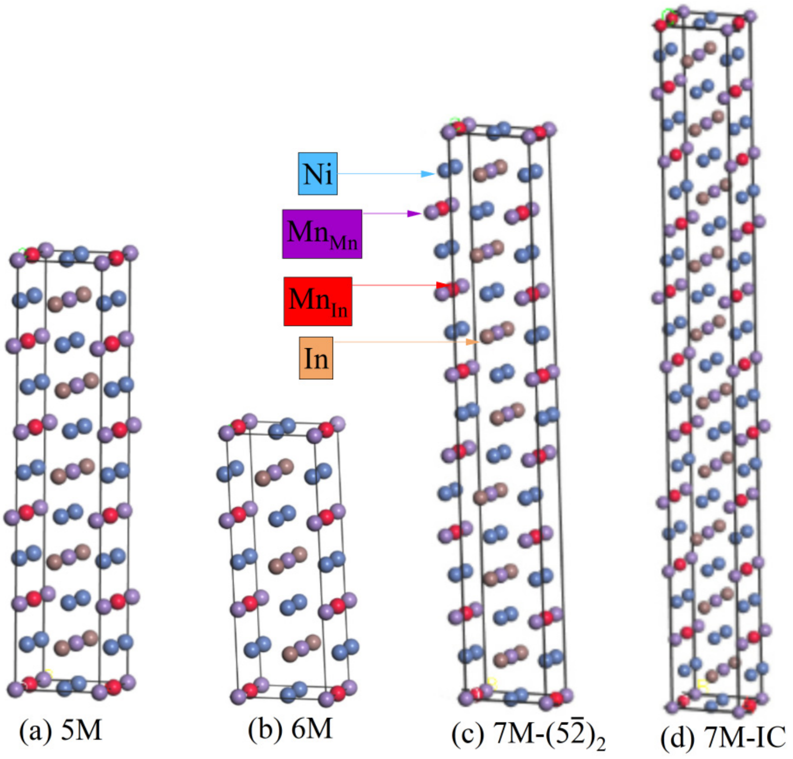

Figure S1. Crystal structures of modulated martensites in $Ni_2Mn_{1.5}In_{0.5}$ alloy. 5M, (b) 6M, (c) 7M-$(5\bar{2})_2$, (d) 7M-IC.

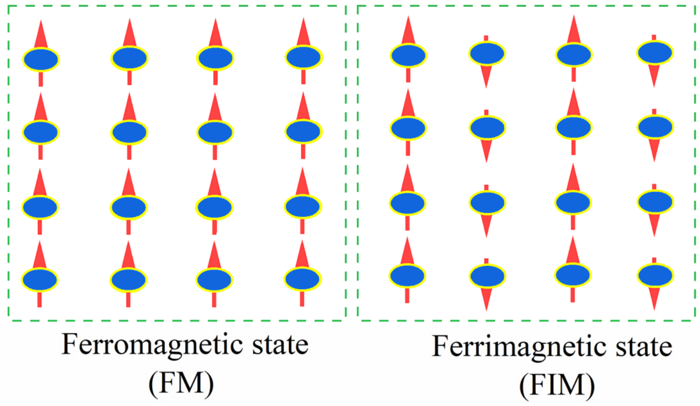

Figure S2. Schematic diagram of the ferromagnetic state (FM) and ferrimagnetic state (FIM).

Table S1. Fractional coordinates of the $Ni_2Mn_{1.5}In_{0.5}$ alloy as derived from the 5M modulated structure. Initial lattice constants: a = 4.226Å; b = 5.581Å; c = 21.052Å; β = 90.3°; Space group: *P2/m* (*No.* 10) [1].

Atom Type	Wyck. position	x	y	z
Ni1	*2j*	1/2	1/4	0
Ni2	*4o*	0.052	1/4	0.1
Ni3	*4o*	0.544	1/4	0.2
Ni4	*4o*	0.956	1/4	0.3
Ni5	*4o*	0.448	1/4	0.4
Ni6	*2k*	0	1/4	1/2
Mn1	*1a*	0	0	0
Mn2	*1h*	1/2	1/2	1/2
Mn3	*2n*	0.552	1/2	0.1
Mn4	*2n*	0.456	1/2	0.3
Mn5	*2m*	0.948	0	0.4
Mn6	*2m*	0.044	0	0.2
MnIn1	*1b*	0	1/2	0
MnIn2	*2n*	0.044	1/2	0.2
MnIn3	*2n*	0.948	1/2	0.4
In1	*2m*	0.552	0	0.1
In2	*2m*	0.456	0	0.3
In3	*1g*	1/2	0	1/2

Table S2. Fractional coordinates of the $Ni_2Mn_{1.5}In_{0.5}$ alloy as derived from the 6M modulated structure. Initial lattice constants: a = 4.3919Å; b = 5.62Å; c = 12.9947Å; β = 93.044°; Space group: *P2/m* (*No.* 10) [2].

Atom Type	Wyck. position	x	y	z
Ni1	*2j*	1/2	1/4	0
Ni2	*2k*	0	1/4	1/2
Ni3	*4o*	0.458	1/4	0.668
Ni4	*4o*	0.111	1/4	0.168
Mn1	*1a*	0	0	0
Mn2	*1h*	1/2	1/2	1/2
Mn3	*2m*	0.0416	1/2	0.3319
Mn4	*2n*	0.611	1/2	0.168
MnIn1	*1b*	0	1/2	0
MnIn2	*2n*	0.0416	1/2	0.3319
In2	*2m*	0.611	0	0.168
In3	*1g*	1/2	0	1/2

Table S3. Fractional coordinates of the $Ni_2Mn_{1.5}In_{0.5}$ alloy as derived from the 7M-IC modulated structure. Initial lattice constants: a = 4.2651Å; b = 5.5114Å; c = 42.365Å; β = 93.27°; Space group: *P2/m* (*No.* 10) [3].

Atom Type	Wyck. position	x	y	z
Ni1	*2l*	1/2	3/4	1/2
Ni2	*2j*	1/2	3/4	0
Ni3	*4o*	0.1056	1/4	0.0501
Ni4	*4o*	0.0075	1/4	0.1502
Ni5	*4o*	-0.092	1/4	0.2496
Ni6	*4o*	0.0511	1/4	0.3499
Ni7	*4o*	0.0395	1/4	0.4505
Ni8	*4o*	0.5663	3/4	0.1004
Ni9	*4o*	0.4786	3/4	0.1996
Ni10	*4o*	0.4151	3/4	0.3000
Ni11	*4o*	0.6055	3/4	0.4002
Mn1	*1a*	0	0	0
Mn2	*1c*	0	0	1/2
Mn3	*2m*	0.0660	0	0.1004
Mn4	*2m*	-0.0214	0	0.1996
Mn5	*2m*	-0.0848	0	0.3000
Mn6	*2m*	0.8945	0	0.5998
Mn7	*2n*	0.6050	1/2	0.0501
Mn8	*2n*	0.5083	1/2	0.1502
Mn9	*2n*	0.4096	1/2	0.2496
Mn10	*2n*	0.5475	1/2	0.3499
Mn11	*2n*	0.5414	1/2	0.4505
MnIn1	*1f*	0	1/2	1/2
MnIn2	*2n*	-0.0214	1/2	0.1996
MnIn3	*2n*	0.9152	1/2	0.3000
MnIn4	*2n*	0.0660	1/2	0.1004
MnIn5	*2m*	0.1055	1/2	0.4002
MnIn6	*1b*	0	1/2	0
In7	*2m*	0.4096	0	0.2496
In8	*2m*	0.5083	0	0.1502
In9	*2m*	0.6050	0	0.0501
In10	*2m*	0.5475	0	0.3499
In11	*2m*	0.5414	0	0.4505

Table S4. Fractional coordinates of the $Ni_2Mn_{1.5}In_{0.5}$ alloy as derived from the 7M-$(5\bar{2})_2$ modulated structure. Initial lattice constants: *a* = 4.257Å; *b* = 5.486Å; *c* = 29.446Å; *β* = 94.2°; Space group: *PM* (*No.* 6) [4].

Atom Type	Wyck. position	x	y	z
Ni1	*2c*	1/2	1/4	0
Ni2	*2c*	-0.0482	1/4	0.0714
Ni3	*2c*	0.4035	1/4	0.1429
Ni4	*2c*	0.1447	1/4	0.2143
Ni5	*2c*	0.3070	1/4	0.2857
Ni6	*2c*	-0.2412	1/4	0.3571
Ni7	*2c*	0.3794	1/4	0.4286
Ni8	*2c*	0	1/4	1/2
Ni9	*2c*	0.4517	1/4	0.5714
Ni10	*2c*	-0.0965	1/4	0.6429
Ni11	*2c*	0.3553	1/4	0.7143
Ni12	*2c*	-0.1930	1/4	0.7857
Ni13	*2c*	0.2588	1/4	0.8571
Ni14	*2c*	-0.1206	1/4	0.9286
Mn1	*1a*	0	0	0
Mn2	*1b*	0.4518	1/2	0.0714
Mn3	*1a*	-0.0965	0	0.1429
Mn4	*1b*	0.3553	1/2	0.2143
Mn5	*1a*	-0.1930	0	0.2857
Mn6	*1b*	0.2588	1/2	0.3571
Mn7	*1a*	-0.1206	0	0.4286
Mn8	*1b*	1/2	1/2	1/2
Mn9	*1a*	-0.0482	0	0.5714
Mn10	*1b*	0.4035	1/2	0.6429
Mn11	*1a*	-0.1447	0	0.7143
Mn12	*1b*	0.3070	1/2	0.7857
Mn13	*1a*	-0.2412	0	0.8571
Mn14	*1b*	0.3794	1/2	0.9286
MnIn1	*1b*	0	1/2	0
MnIn2	*1b*	-0.0965	1/2	0.1429
MnIn3	*1b*	-0.1930	1/2	0.2857
MnIn4	*1b*	-0.1206	1/2	0.4286
MnIn5	*1b*	-0.0482	1/2	0.5714
MnIn6	*1b*	-0.1447	1/2	0.7143
MnIn7	*1b*	-0.2412	1/2	0.8571
In1	*1a*	0.4518	0	0.0714
In2	*1a*	0.3553	0	0.2143
In3	*1a*	0.2588	0	0.3571
In4	*1a*	1/2	0	1/2
In5	*1a*	0.4035	0	0.6429
In6	*1a*	0.3070	0	0.7857
In7	*1a*	0.3794	0	0.9286

Reference

[1] Glavatskyy I, Glavatska N, Urubkov I, Hoffman J U, Bourdarot F, 2008 *Mate. Sci. Eng., A* **481-482** 298-301.
[2] Yan H, Zhang Y, Xu N, Senyshyn A, Brokmeier H G, Esling C, Zhao X, Zuo L, 2015 *Acta Mater.* **88** 375-388.
[3] Righi L, Albertini F, Villa E, Paoluzi A, Calestani G, Chernenko V, Besseghini S, Ritter C, Passaretti F. 2008 *Acta Mater.* **56** 4529-4535.
[4] Z.B. Li, Study on crystallographic features of Ni-Mn-Ga ferromagnetic shape memory alloys, Dissertation for Doctor's Degree. Paul-Verlaine university of Metz and Northeastern University, 2011.
